# A high-efficiency trichome collection system by laser capture microdissection

**DOI:** 10.3389/fpls.2022.985969

**Published:** 2022-08-22

**Authors:** Wei Qin, Yongpeng Li, Bowen Peng, Hang Liu, Tiantian Chen, Xin Yan, Yaojie Zhang, Chen Wang, Xinghao Yao, Xueqing Fu, Ling Li, Kexuan Tang

**Affiliations:** Frontiers Science Center for Transformative Molecules, Joint International Research Laboratory of Metabolic and Developmental Sciences, Key Laboratory of Urban Agriculture (South) Ministry of Agriculture, Plant Biotechnology Research Center, Fudan-SJTU-Nottingham Plant Biotechnology R&D Center, School of Agriculture and Biology, Shanghai Jiao Tong University, Shanghai, China

**Keywords:** *Artemisia annua* L., glandular secretory trichome, artemisinin, laser capture microdissection, secondary metabolites

## Abstract

Trichomes, which are classified as glandular or non-glandular, are hair-like epidermal structures that are present on aerial parts of most plant species. Glandular secretory trichomes (GSTs) have the capacity to secrete and store specialized metabolites, which are widely used as natural pesticides, food additives, fragrance ingredients or pharmaceuticals. Isolating individual trichomes is an essential way for identifying trichome-specific gene functions and discovering novel metabolites. However, the isolation of trichomes is difficult and time-consuming. Here, we report a method to isolate the GSTs from leaf epidermis dispense with fixation using laser capture microdissection (LCM). In this study, 150 GSTs were captured efficiently from *Artemisia annua* leaves and enriched for artemisinin measurement. UPLC analysis of microdissected samples indicated specific accumulation of secondary metabolites could be detected from a small number of GSTs. In addition, qRT-PCR revealed that the GST-specific structural genes involved in artemisinin biosynthesis pathway were highly expressed in GSTs. Taken together, we developed an efficient method to collect comparatively pure GSTs from unfixed leaved, so that the metabolites were relatively obtained intact. This method can be implemented in metabolomics research of purely specific plant cell populations and has the potential to discover novel secondary metabolites.

## Introduction

Trichomes are the special structures derived from the epidermal cell of plant aerial part (Werker, 2000). Nearly 30% of all vascular plants have multicellular glandular trichomes (Glas et al., 2012). Because of the different secondary metabolism abilities, trichomes are segmented into non-glandular and glandular trichomes (Serna and Martin, 2006; Huchelman et al., 2017). Two types of trichomes are covered on both the adaxial and abaxial leaf surfaces of *Artemisia annua*, T-shaped trichomes (TSTs) and GSTs, and GST is identified as the main spot for artemisinin synthesis and accumulation (Ascensao and Pais, 1987; Duke and Paul, 1993). Besides, characteristic essential oil and various of potential secondary metabolites including flavonoids, phenolics, and terpenoids/terpenes may be accumulated in GSTs (Bhakuni et al., 2001; Ahmad Malik et al., 2009; Ferreira et al., 2010). Accordingly, GSTs are considered to be intriguing natural products troves with substantial economic potential for medicines, essential oils, and natural insecticide, where numerous known or undetected metabolites and metabolic pathways can be discovered (Glas et al., 2012; Sallets et al., 2014; Huchelman et al., 2017).

It is feasible to directly harvest trichomes from plants only adhering single type of trichome by brushing the plant surfaces, shaking the leaves or buds in aqueous solution, or brushing the frozen leaves (McCaskill et al., 1992; Wang et al., 2001; Feng et al., 2021). However, in most cases, multiple types of trichome appear simultaneously in plant. Thus, these approaches are not suitable for them. In some species, like *A. annua*, the stalk cells of GSTs are firmly stuck on the epidermis, shaking buds in aqueous solution might be the only feasible way to obtain enough GSTs, while they are still mixed with TSTs and limited by the floral bud stage (Wang et al., 2009). In tomato, it is able to isolate trichomes by brushing the leaves (Balcke et al., 2014), but subsequent selection of each type is time-consuming, and laser capture microdissection (LCM) is required.

Laser capture microdissection is an extraordinary technology using microscope and laser to harvest specific populations of tissue cells and even organelles (Day et al., 2005; Espina et al., 2006). The collected samples need proper fixation which is one of the most critical steps during sample preparation for LAM and then they can be used to extract DNA, RNA, or protein for genomic, transcriptomic, proteomic studies of specific cell types (Kerk et al., 2003; Nakazono et al., 2003; Day et al., 2005; Fang and Schneider, 2014). In plants, it has been broadly used to explore the gene expression in different tissues (Hacquard et al., 2010). To date, however, LCM used for metabolic analysis has been rarely reported because of the limitation by the use of various fixatives (Gautam and Sarkar, 2015). The fixation is not helpful for tissue dedicated to the analysis of small molecules which can be soluble in the solvent used (Hölscher and Schneider, 2008). In addition, the fixative-free procedure of cryosectioning gave the only chance to exclude the total extraction of secondary metabolites (Hölscher and Schneider, 2008). Nevertheless, the large vacuoles may cause the loss of integrity of plant cells (Nelson et al., 2006). Thus, in order to isolate high-quality single type of trichomes from live plants without fixation or cryosections for deep studies, an improved method is needed.

In this study, we developed an improved strategy for LCM without tissue fixation or cryosections to capture unitary and purer trichomes. 150 high-quality GSTs of *A. annua* were captured efficiently in 30 min and used for artemisinin measurement using UPLC/IM-QTOF-HDMS. The artemisinin and arteannuin B were detected clearly, and 15 potential compounds in GSTs were identified. This improvement may be used to exploit the studies on plant metabolic profiles, as well as protein analyses.

## Materials and methods

### Plant materials

The *A. annua* plants used in this study was “Huhao 1” originated from Chongqing, China and subjected to several years of selection in Shanghai (Shen et al., 2016). *A. annua*, *Solanum lycopersicum* (Var. Micro-Tom) and *Nicotiana benthamiana* were cultured in greenhouse under a 16 h/8 h light/dark photoperiod at 25 ± 2°C. Leaves of 6-month-old *A. annua* wild type plants were used in this study. *S. lycopersicum* and *N. benthamiana* wild type plants used in this study were 6–8-week-old. For *A. annua*, the leaf8 was picked for following experiments.

### Observation of trichomes

The mature leaves (leaf8, the eighth leaf below meristem) of *A. annua* plants grown in the glasshouse were selected for the observation of glandular trichomes. The morphological characteristics of GSTs were observed using an Olympus BX51 light microscope (Olympus, Tokyo, Japan) under white light, blue light, and UV. Images were taken using a digital camera attached to the microscope.

### Preparation for LCM

**Figure 1:**
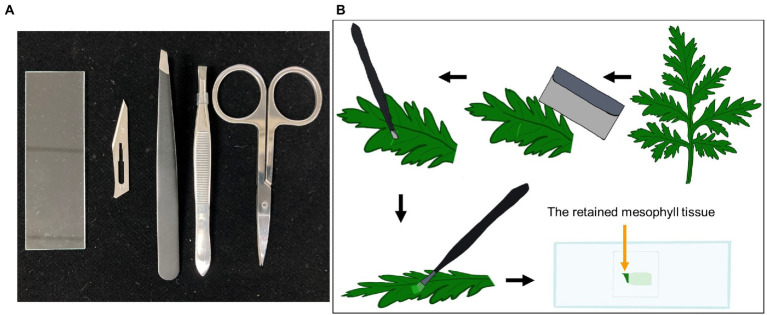
**(A)** Tool kit including precise slanted tweezer, surgical knife blade, scissor, and glass slide for tissue sample preparation; **(B)** Schematic representation for the process of tearing off epidermis from the odd-pinnately compound leaf of *Artemisia annua*. The first step in the process was to select healthy fresh leaves. Secondly, gently scratch the leaves with surgical blade. Thirdly, tear off the uniseriate epidermis using slanted tweezer softly, with a little of mesophyll tissue retained. The final step was to spread the epidermis onto PEN slides for the subsequent experiment.

The cap of a 0.5 ml Eppendorf® RNA/DNA LoBind tube was filled with ~50 μl 0.01% agar solution for to catch the captured samples. Precise slanted tweezer, surgical knife blade, scissor, and glass slide were used as a tool kit to tear off the plant epidermis (Figure 1A).

### Laser capture microdissection system

Laser Capture Microdissection (LCM) was performed using the PALM® Laser-Microbeam system (Carl Zeiss Meditec AG, Jena, Germany). The PALM® system includes computer-controlled X–Y stage, an inverted microscope with attached color CCD camera, and LCM capture unit. LCM was performed on polyethylene naphthalate (PEN) membrane slides (Carl Zeiss Meditec AG, Jena, Germany).

For cut energy, the optimal value we found was 54 and for laser pressure catapult (LPC) energy the best value was 60. The best values were 70 for cut focus, and the optimal value for LPC focus was 46. Because the cell density and species vary, these values may be changed appropriately. It is recommended to explore the optimal values when changing species.

### Sample extraction

The collected GSTs were extracted by 200 μl methanol, and treated ultrasonically twice (55 HZ, 30 min). Then, the extract was evaporated to dryness in the Thermo Savant SPD 2010 Speed-Vac System (Thermo Electron Corporation, United States). The residue was immediately reconstituted in 70 μl methanol. The concentrations of artemisinin and arteannuin B were 250 ng/ml and 200 ng/mL, respectively.

### Artemisinin detection

The artemisinin detection was performed on an ACQUITY UPLC I-Class/Vion IMS-QTOF system (Waters, Milford, MA, United States). To identify artemisinin and arteannuin B in extracts, standards were used to obtain the retention time, extracted ion chromatogram (EIC), mass-to-charge (m/z) ratios.

A BEH Shield C18 column (2.1 × 100 mm, 1.7 μm) maintained at 45°C was used for chromatographic separation. A binary mobile phase consisting of 0.1% FA in CH3CN (organic phase: A) and 0.1% FA in H2O (water phase: B) was employed at a flow rate of 0.4 ml/min following an optimized gradient program: 0–3 min, 95–80% (A); 3–10 min, 80–0% (A); 10–12 min, 0% (A); 12–15 min, 0–5% (A); and 15–19 min, 5% (A). Each 1 μl of the sample solution was used for injection.

The data were acquired by a Vion IMS-QTOF mass spectrometer in the ESI positive/negative MS^E^ mode (Waters Corporation). The LockSpray ion source was equipped under the following parameters: capillary voltage, 2.0 kV (positive/negative); cone voltage, 40 V; desolvation temp, 450°C; desolvation gas, 900 l/h; Cone gas, 50 l/h; source temperature, 115°C; acquisition range, 50 to 1,000 m/z; scan rate, 0.2 s; collision energy, 6 eV/20 ~ 45 eV. The locking mass calibration Tyr-Gly-Gly-Phe-Leu (leucine-encephalin; flow rate, 10ul/min) was used as the lock mass. Data acquisition and processing were performed by the UNIFI™ 1.9.3.0 software (Waters). The accuracy error threshold was fixed at 10 ppm.

### RNA extraction and quantitative real-time PCR

Total RNA of captured GSTs and epidermal cells was extracted using the RNA prep Pure Micro Kit (Tiangen, Beijing, China), following the manufacturer’s instructions. The RNA samples were reverse transcribed into cDNA using the PrimeScript II RT Master Mix (Takara, Dalian, China). qRT–PCR analysis was performed and conducted as described previously (Shen et al., 2016). Three independent experiments for each sample were performed. All the primers sequences used in qRT-PCR are listed in Supplementary Table S1.

## Results

### Consideration factors in sample preparation

**Figure 2:**
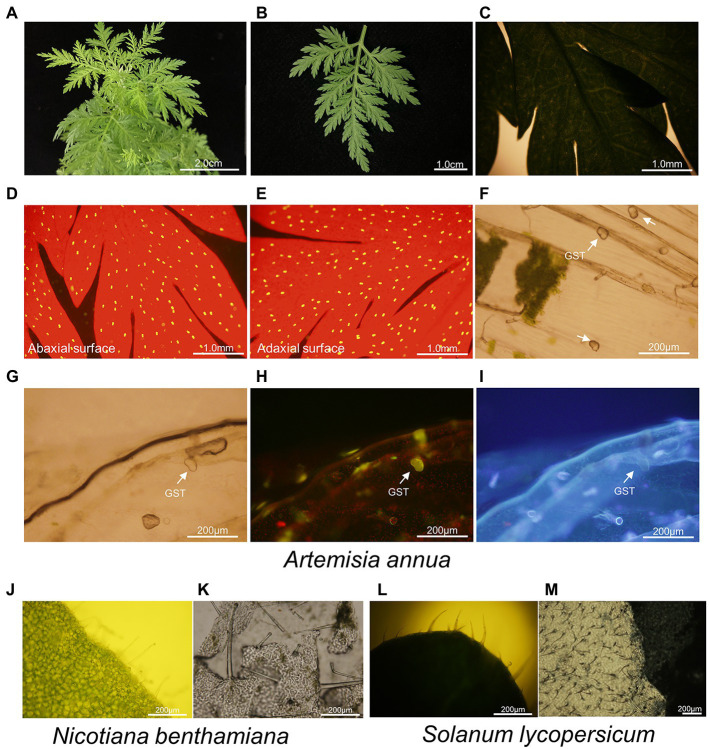
Leaf and trichome morphologies of *A. annua*, *N. benthamiana* and *S. lycopersicum*. **(A–C)** Overview of odd-pinnately compound leaf of 6-month-old *A. annua* plant; **(D–I)** Microscopic images of trichomes of *A. annua*. **(D,E)** Abaxial leaf surface and adaxial leaf surface were observed under blue light, with red backgrounds representing chlorophyll and yellow spots representing glandular secretory trichomes; Uniseriate epidermis was observed under **(F,G)** white light, **(H)** blue light, and **(I)** UV. Images were taken using a BX-51 microscope (Olympus, Tokyo, Japan). The glandular secretory trichomes were marked with white arrows; **(J)** Microscopic images of trichomes on the leaf of *N. benthamiana*; **(K)** Microscopic images of uniseriate epidermis of *N. benthamiana*; **(L)** Microscopic images of trichomes on the leaf of *S. lycopersicum*; **(M)** Microscopic images of uniseriate epidermis of *S. lycopersicum*.

The leaves of *A. annua* are small, very soft to touch and odd-pinnately compound with deeply indented margins (Figures 2A–C; Alejos-Gonzalez et al., 2011; Li et al., 2021). That is to say, operating on the narrow leaves of *A. annua* directly and precisely seems to be unfulfillable. On the other hand, a plenty of GSTs and TSTs are firmly anchored on both the adaxial and abaxial leaf surfaces (Figures 2D–I). However, the thickness of *A. annua* leaves prevented observation of neither GSTs nor TSTs under white light (Figure 2C). Due to the multiple trichome types on both sides of leaves and the thickness of leaves, it is more difficult to directly collect a single type of trichome. Hence, tearing off the epidermis of leaves makes it possible to precisely dissect and collect individual GSTs population without mesophyll tissue or another type of trichomes (Figure 2F).

Likewise, we observed the leaves of *N. benthamiana* and *S. lycopersicum*, both of them contained multiple types of trichomes (Figures 2J,L). It was hard to directly observe the trichomes limited by the thickness of leaves, while they were easily distinguishable on a layer of epidermis (Figures 2J–M).

### Laser capture microdissection of GSTs of *Artemisia annua*

To peel off the basal layer of the epidermis, we improved a process (Figure 1B). The first step was to select healthy fresh leaves from 6-month-old *A. annua* plants. Secondly, the untreated fresh leaf of *A. annua* was gently scratched by surgical blade, it should be noted that the leaves cannot be pierced through. Then, the epidermis began to be torn off from the scratch using slanted tweezer softly. At last, the epidermis was spread onto PEN slides for subsequent LCM. Notably, since the extremely thin layer of epidermis was easy to be curled, a little of mesophyll tissue was supposed to be retained.

**Figure 3:**
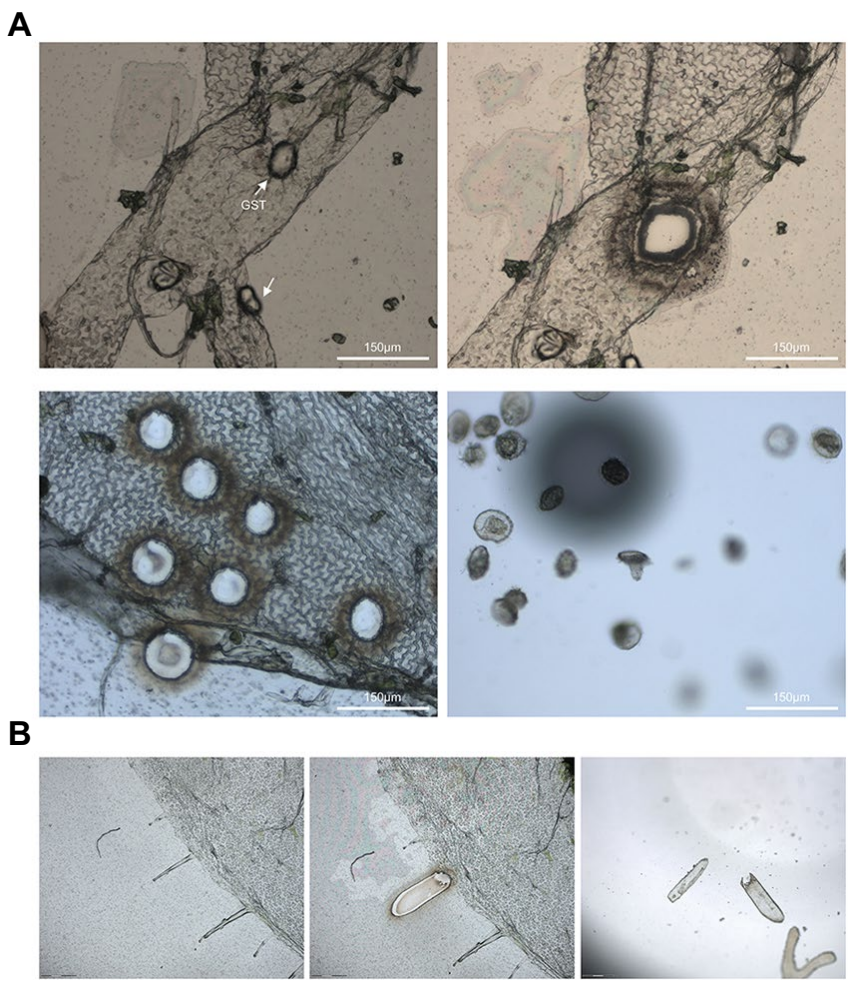
**(A)** The glandular secretory trichomes of *A. annua* was selected using the PALM® Laser-Microbeam system. The optimal value for cut energy was 54 and the best value for laser pressure catapult energy was 60. The best values were 70 for cut focus, and the optimal value for LPC focus was 46. **(B)** Laser capture microdissection of glandular secretory trichomes of *N. benthamiana*.

The GSTs was precisely collected on the cap of 0.5 ml microcentrifuge tube using LCM system (Figure3A). Total of 150 GSTs were captured in a tube in 30 min. This result indicated that the modified method was time-saving and high-efficiency to collect pure GSTs.

Furthermore, the epidermises of *N. benthamiana* and *S. lycopersicum* were peeled off following the same process, and the epidermis could be used for LCM as well (Figure 3B). This results confirmed that the LCM combining with tearing epidermis is a general approach to gathering trichomes or other distinct cell types for many plant species indeed.

### Artemisinin detection using UPLC/IM-QTOF-HDMS

**Figure 4:**
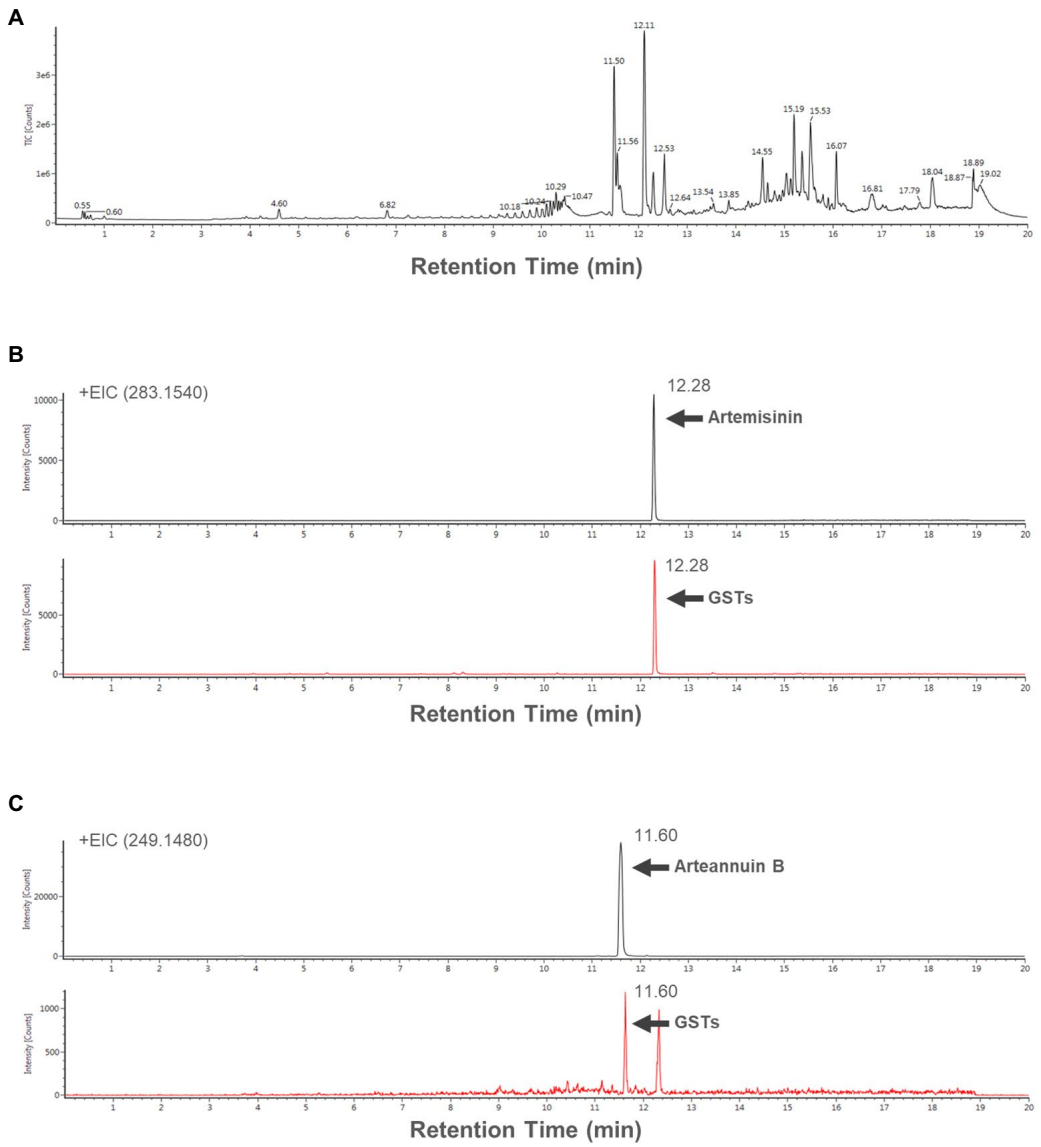
**(A)** Total ion chromatogram (TIC) of the extract form 150 GSTs. Extracted ion chromatograms (EICs) of representative samples **(B)** artemisinin [283.1540 (*m*/*z*)^+^], and **(B)** arteannuin B [249.1480 (*m*/*z*)^+^].

The 150 captured GSTs were used to extract the contents and analyzed the compounds with UPLC/IM-QTOF-HDMS. Total ion chromatogram (TIC) of GSTs extract is shown in Figure 4A. The artemisinin and arteannuin B, which are specifically accumulated in GSTs, were detected with a very clear peak (Figures 4B,C).

Using UPLC-QTOF/MS analysis combined with the database available in the UNIFI system, 15 potential compounds of GSTs were identified, and the detailed information of the 15 compounds is listed in Table 1. In general, these results demonstrated that it was possible to extract and detect specific or potential compounds from only 150 GSTs.

**Table 1:** The information of 15 potential compounds in GSTs based on UHPLC-Q-TOF with the database available in the UNIFI system.

**Component name**	**Formula**	**Observed RT (min)**	**Neutral mass (Da)**	**Observed neutral mass (Da)**	**Observed m/z**	**Mass error (ppm)**
2-Monopalmitin	C19H38O4	11.32	330.27701	330.2794	348.3133	7
Valine	C5H11NO2	5.52	117.07898	117.0792	118.0865	2.2
7-Methoxycoumarin	C10H8O3	4.93	176.04734	176.0474	177.0546	0
4-Feruloylquinic acid	C17H20O9	4.56	368.11073	368.1114	391.1007	1.8
Linolein	C57H98O6	17.99	878.73634	878.7376	879.7449	1.4
6-Methoxyl-7-hydroxycoumarin	C10H8O4	5.14	192.04226	192.0423	193.0496	0.2
Pachymic acid	C33H52O5	14.36	528.38147	528.3798	529.3871	−3.1
Chlorogenic acid	C16H18O9	3.92	354.09508	354.0948	377.084	−0.7
Phosphatidyl ethanolamines	C41H80NO8P	14.85	745.56216	745.5644	746.5717	3
Eupatin	C18H16O8	10.48	360.08452	360.0865	361.0937	5.4
Phenethyl ferulate	C18H18O4	12.3	298.12051	298.1221	321.1113	4.9
Heterophyllin A	C37H57N7O8	16.8	727.42686	727.4272	750.4164	0.4
Casticin	C19H18O8	11.49	374.10017	374.1029	375.1101	7.2
Farnesiferol A	C24H30O4	12.12	382.21441	382.2167	400.2505	5.6
Ivalin	C15H20O3	16.07	248.14124	248.142	266.1758	2.8

### Expression analyses of structural genes involved in artemisinin biosynthesis

**Figure 5:**
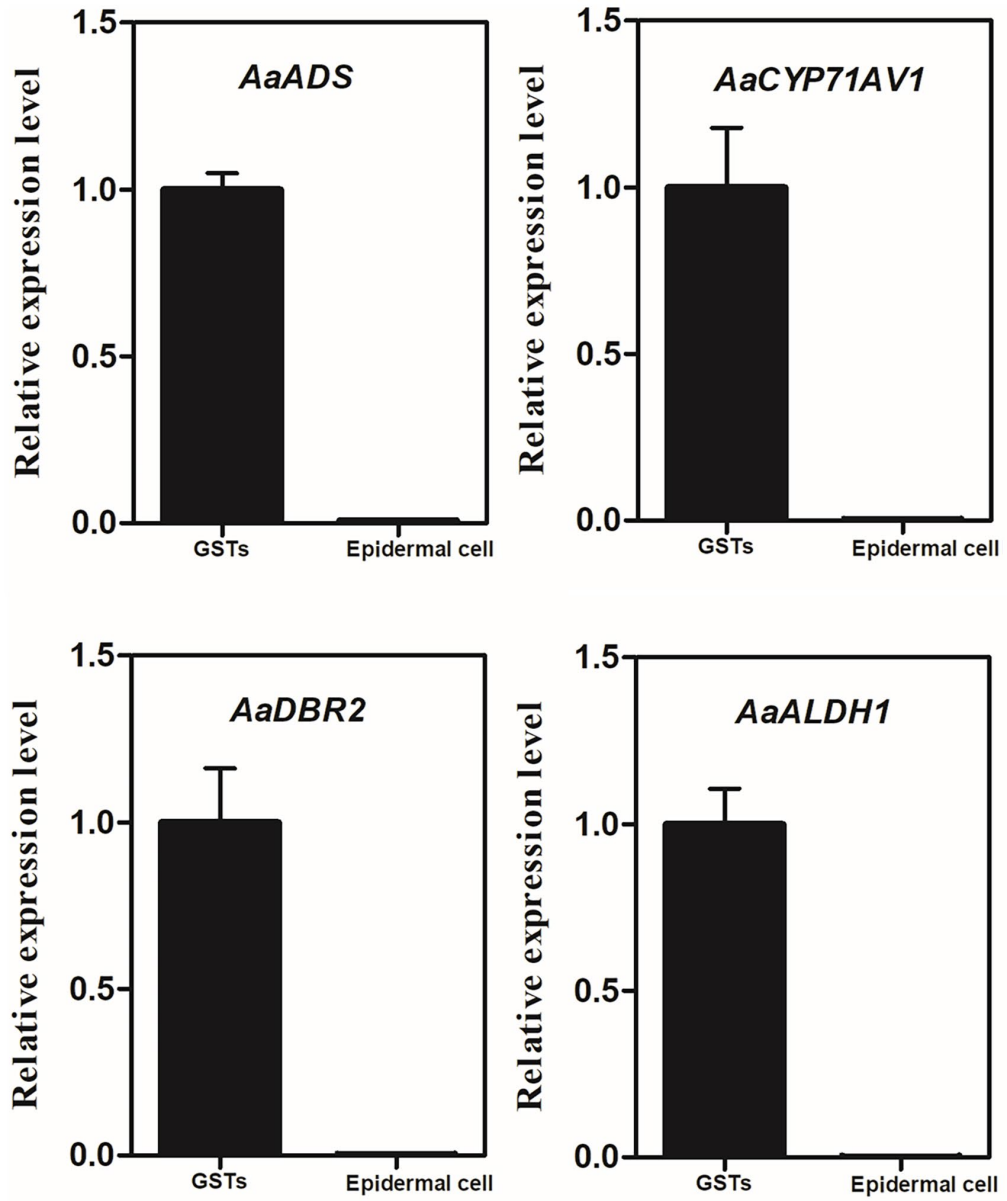
Relative expression of *AaADS*, *AaCYP71AV1*, *AaDBR2* and *AaALDH1* in GSTs and epidermal cells of *A. annua* by quantitative real-time polymerase chain reaction. β-actin was used as an internal control. Error bars represent the standard deviation (*n = 3*).

Quantitative real-time PCR was performed using cDNA from the captured GSTs and the epidermal cells to analyze the transcript level of *AaADS*, *AaCYP71AV1*, *AaDBR2* and *AaALDH1*, which are the GST-specific genes involved in artemisinin biosynthesis (Figure 5). The results indicated that these four genes were highly expressed in GSTs.

## Discussion

Researches have shaped our understanding of the multitudinous secondary metabolites in glandular trichomes over the past few decades. In *Mentha spicata* L., two types of glandular trichomes, capitate and peltate were observed (Champagne and Boutry, 2013). Volatile essential oils as secondary metabolites are mostly produced in peltate glandular trichomes (PGTs), including some monoterpenes, limonene and carvone (Alonso et al., 1992; Champagne and Boutry, 2013; Markus Lange and Turner, 2013; Wang et al., 2016). There are five types of trichomes on *Cannabis sativa* L., three of them are glandular trichomes, namely capitate-stalked, capitate-sessile, and bulbous trichomes (Dayanandan and Kaufman, 1976; Happyana et al., 2013). The glandular trichomes are as the main production and storage site to terpenes, and cannabinoids which is famous for their psychoactive and therapeutic effects (Kim and Mahlberg, 1991; Mahlberg and Kim, 2004). Two types of trichomes are described on the leaves of tobacco, the short type may secret hydrophilic nicotine, while the long type produces a resin containing diterpene cembratrienediol (Meyberg et al., 1991; Amme et al., 2005). There are multiple trichomes classified as types I–VII in tomato (*S. lycopersicum*). Types I, IV, VI and VII are glandular trichomes, whereas types II, III and V are non-glandular trichomes (Jin-Ho et al., 2010). Type I and type IV trichomes are involved in acyl sugar biosynthesis, while type VI trichomes are considered as the foremost spot for the secretion of terpenoid, flavonoid and methyl ketone (Bleeker et al., 2011; Feng et al., 2021). Therefore, glandular trichomes of plants are considered as a great site for secreting and storing abundant specialized secondary metabolites including terpenoids/terpenes, phenylpropanoids, flavonoids, methyl ketone, polyphenols, and acyl sugars, and these compounds can be broadly used as natural medications, insect repellents or essential oils (Tattini et al., 2000; Dixon, 2001; Fridman et al., 2005; Gershenzon and Dudareva, 2007; Schilmiller et al., 2008; Tissier, 2012; Huchelman et al., 2017). So that understanding the high-efficiency metabolic network of glandular trichomes and the transport and storage of metabolites could be the next challenge (Schuurink and Tissier, 2020).

Laser capture microdissection (LCM) is an advanced technology that allows the identification, selection, and isolation of pure cell populations. Generally speaking, LCM requires histological processing and fixation to stabilize proteins and RNA, nevertheless, this procedure using various fixatives, coagulative fixatives such as acetone and alcohol or cross-linking fixatives such as formaldehyde, may wash out or damage the metabolites (Gillespie et al., 2002; Inada and Wildermuth, 2005; Schad et al., 2005; Schneider and Hlscher, 2007; Li et al., 2012; Fang and Schneider, 2014; Goldsworthy et al., 2015). Moreover, using cryosections can extract RNA, proteins, and metabolites without histological fixation (Asano et al., 2002; Nakazono et al., 2003; Schad et al., 2005, 2010; Li et al., 2012). However, unlike animal cells, plant cells will lose their integrity in cryosections as a result of the large vacuoles and cell wall of variable rigidity (Nelson et al., 2006; Gautam and Sarkar, 2015). Besides, in RNA analysis, adequate RNA samples for studies can be achieved by linear amplification (Kerk et al., 2003; Nelson et al., 2006). But this amplification is not suitable for proteins and metabolites, which are limited by the abundance of cellular targets (Fang and Schneider, 2014). So that, it is necessary to develop a method dispense with tissue fixation or cryosections.

Comparing to the traditional way using brushes, LCM is a more direct way to isolate pure trichomes. Accordingly, LCM of plant trichomes is applied in some cases. In *A. annua*, LCM was used to isolate different whole cells of *A. annua* GSTs from slices of leaves and flower buds fixing with 4% para-formaldehyde. While trichomes perpendicular to the laser beam are necessary, which increase the randomness and uncontrollability (Olsson et al., 2009). In *C. sativa*, capitate-sessile and capitate-stalked trichomes were isolated over the flowering period using LCM with neither fixation nor freeze (Happyana et al., 2013). In *Colquhounia coccinea*, peltate glandular trichomes was collected directly from unfixed leaves using LCM (Li et al., 2013). However, the obtained trichomes were not trichome-exclusive and always adhered with redundant mesophyll. On the other hand, a special angle of trichomes is needed. So far, capturing unfixed trichomes or other plant cells is still difficult and costly.

Strikingly, the method we described in this study provides a very instructive solution to shape the metabolic network of plant trichomes. In our study, the extract of 150 GSTs provided a very clear peak and strong signal of artemisinin, and offered a number of potential compounds using database available. In addition, the expression level of *AaADS*, *AaCYP71AV1*, *AaDBR2* and *AaALDH1* (Figure 5), which are the key structural genes involved in the artemisinin biosynthesis pathway, verified the reliability of our method. Accordingly, collecting more numerous GSTs may get stronger signals and more information. That is to say, it can give us insights into the known or undiscover molecules with bioactive potential in plant GSTs.

In conclusion, we have provided an alternative and promising strategy, which is suitable for many other plant species to efficiently capture trichomes, to isolate GSTs without fixation by LCM. 150 GSTs of *A. annua* were captured in 30 min and used for metabolites analysis using UPLC/IM-QTOF-HDMS. The artemisinin and arteannuin B were detected, respectively, and 15 potential compounds in GSTs were identified. This improvement may offer the possibility of trichome-specific proteomics and metabolomics, or other single cell analysis.

## Data availability statement

The original contributions presented in the study are included in the article/Supplementary material, further inquiries can be directed to the corresponding author.

## Author contributions

WQ and KT designed the research. WQ, YL, BP, HL, TC, XY, YZ, CW, and XGY performed most of the experiments. WQ and YL drafted the manuscript. XF, LL, and KT revised the manuscript. All authors contributed to the article and approved the submitted version.

## Funding

This work was supported by National Key R&D Program of China (2018YFA0900600), the Bill & Melinda Gates Foundation (OPP1199872), the National Natural Science Foundation of China (31770327), SJTU Trans-med Awards Research (20190104) and SJTU Global Strategic Partnership Fund (2020 SJTU-CORNELL).

## Conflict of interest

The authors declare that the research was conducted in the absence of any commercial or financial relationships that could be construed as a potential conflict of interest.

## Publisher’s note

All claims expressed in this article are solely those of the authors and do not necessarily represent those of their affiliated organizations, or those of the publisher, the editors and the reviewers. Any product that may be evaluated in this article, or claim that may be made by its manufacturer, is not guaranteed or endorsed by the publisher.
