# Supplementary material for: A high-efficiency trichome collection system by laser capture microdissection
Source: Front Plant Sci. 2022 Aug 22;13:985969. doi: 10.3389/fpls.2022.985969 (PMC9441851; doi:10.3389/fpls.2022.985969)
Supplement: Supplementary file 1 [file Table_2.DOCX]

***Supplementary Material***

**A high-efficiency trichome collection system by laser capture microdissection**

**Wei Qin, Yongpeng Li, Bowen Peng, Hang Liu, Tiantian Chen, Xin Yan, Yaojie Zhang, Chen Wang, Xinghao Yao, Xueqing Fu, Ling Li, and Kexuan Tang***

***Correspondence:**Kexuan Tang
kxtang@sjtu.edu.cn

Table S1 Primers used in the study

| Primer | Primer Sequence (5'→3') | Purpose |
| --- | --- | --- |
| *AaActin-F* | CCAGGCTGTTCAGTCTCTGTAT | qRT-PCR |
| *AaActin-R* | CGCTCGGTAAGGATCTTCATCA | qRT-PCR |
| *AaADS-F* | AATGGGCAAATGAGGGACAC | qRT-PCR |
| *AaADS-R* | TTTCAAGGCTCGATGAACTATG | qRT-PCR |
| *AaCYP71AV1-F* | CGAGACTTTAACTGGTGAGATTGT | qRT-PCR |
| *AaCYP71AV1-R* | CGAAGCGACTGAAATGACTTTACT | qRT-PCR |
| *AaDBR2-F* | GCGGTGGTTACACTAGAGAACTT | qRT-PCR |
| *AaDBR2-R* | ATAATCAAAACTAGAGGAGTGACCC | qRT-PCR |
| *AaALDH1-F* | AGCGACAGAAGAAGTGTTAGCAA | qRT-PCR |
| *AaALDH1-R* | TAACTCGTCAGCATTTTCATCG | qRT-PCR |
